# Low-Protein Diets, Malnutrition, and Bone Metabolism in Chronic Kidney Disease

**DOI:** 10.3390/nu16183098

**Published:** 2024-09-13

**Authors:** Cidália D. Pereira, Carla Guimarães, Vânia S. Ribeiro, Daniela C. Vaz, Maria João Martins

**Affiliations:** 1 School of Health Sciences, Polytechnic of Leiria, 2411-901 Leiria, Portugal; cidalia.pereira@ipleiria.pt (C.D.P.); carla.guimaraes@ipleiria.pt (C.G.); vania.ribeiro@ipleiria.pt (V.S.R.); daniela.vaz@ipleiria.pt (D.C.V.); 2 Centre for Innovative Care and Health Technology, Polytechnic of Leiria, 2411-901 Leiria, Portugal; 3 Laboratory of Separation and Reaction Engineering-Laboratory of Catalysis and Materials (LSRE-LCM), ESTG-IPLeiria, 2411-901 Leiria, Portugal; 4 ALiCE—Associate Laboratory in Chemical Engineering, University of Porto, 4200-465 Porto, Portugal; 5 Coimbra Chemistry Centre (CQC), Institute of Molecular Sciences, Chemistry Department, University of Coimbra, 3004-535 Coimbra, Portugal; 6 Instituto de Investigação e Inovação em Saúde (i3S), Universidade do Porto, 4200-135 Porto, Portugal; 7 Unit of Biochemistry, Department of Biomedicine, Faculty of Medicine, University of Porto, 4200-319 Porto, Portugal

**Keywords:** chronic kidney disease, diabetes, mineral and bone disorders, low-protein diet, very low-protein diet, keto-analogues, malnutrition

## Abstract

Chronic kidney disease (CKD) has a high prevalence worldwide, with increasing incidence in low- and middle-income countries, and is associated with high morbidity and mortality, particularly from cardiovascular disease. Protein-restricted diets are one of the most widely used non-pharmacological approaches to slow the progression of CKD and prevent associated metabolic abnormalities. However, some concerns have been raised about the long-term safety of these diets, particularly with regard to patients’ nutritional status and bone and mineral disorders. Therefore, the aim of this article is to review the most recent scientific evidence on the relevance of using protein-restricted diets (with or without keto-analogue supplementation) and, in particular, their relationships with malnutrition and mineral and bone disorders in people with CKD without kidney replacement therapies. Although protein-restricted diets, especially when supplemented with keto-analogues and highly personalized and monitored, do not appear to be associated with malnutrition, research on their effects on bone and mineral disorders is scarce, deserving further investigation.

## 1. Introduction

Chronic kidney disease (CKD), with a global prevalence of 9.1%, has become a relevant global health issue, with an important burden in low- and middle-income countries, owing to their limited access to adequate CKD treatments. Kidney disease is now the seventh principal risk factor for mortality worldwide, and it increases cardiovascular disease (CVD)-related morbidity and mortality [1,2].

According to Kidney Disease: Improving Global Outcomes (KDIGO, an international non-profit organization researching and applying evidence-based clinical practice guidelines in this area), CKD is (a) characterized by kidney damage markers or a decreased glomerular filtration rate (GFR) lasting more than 3 months and (b) classified according to cause, GFR, and albuminuria criteria, with implications for patient health [3,4].

Diabetes and/or hypertension are recognized as two important risk factors for CKD. In addition, aging, obesity, dyslipidaemia, glomerulonephritis, infection, environmental exposure (such as smoking or heavy metals), and genetic factors also contribute to the increase in CKD risk [2]. 

After CKD diagnosis, both pharmacological and non-pharmacological approaches are used to retard the progression of the disease and the need for kidney replacement therapies, targeting the etiological factors. Regarding non-pharmacological strategies, diet plays a central role [5,6], traditionally focusing on reducing protein, phosphorus, potassium, and sodium intake to slow the progression of CKD [7,8]. Low-protein diets are among the most used dietary interventions regarding CKD [9,10,11,12], as they improve hyper-filtration and lessen nitrogenous waste, as well as glomerular pressure [7], preventing CKD progression or end-stage chronic kidney disease (ESKD) [13,14]. However, despite the potential benefits of low-protein diets in CKD, there are also some concerns about protein restriction, as some research highlights that it might increase the risk of malnutrition and/or protein–energy wasting (PEW), particularly in elderly patients with CKD [15,16,17]. In addition, the kidneys play an important role in bone health, with alterations in mineral and bone metabolism seen when renal function declines [18]. Considering that, the aim of this article is to review the most recent scientific evidence on the relevance of using protein-restricted diets and, in particular, their relationships with malnutrition and mineral and bone disorders, in people with CKD without kidney replacement therapies.

## 2. Low and Very Low-Protein Diets: Definition and Rationale for Their Use

Regarding protein intake recommendations, the Institute of Medicine has established a recommended dietary allowance of 0.80 g of protein/kg of body weight/day for sedentary, healthy adults [19]. In the European context, the European Food Safety Authority (EFSA) has published population reference intakes (PRIs) for protein. A PRI indicates the amount of an individual nutrient that the majority of people in a population needs for good health, depending on their age and sex. For adults (including older adults), a PRI of 0.83 g of protein/kg of body weight/day has been established [20]. However, the average protein intake in many Western countries is 150–200% of recommended values, and in Europe, the total per capita protein consumption (including vegetable sources) is about 70% higher than recommended [21]. Moreover, there is some evidence supporting adverse effects of long-term high-protein diets (above the recommendations and particularly from animal origin), namely disorders of bone and calcium homeostasis and of renal and liver function, as well as increased cancer risk and accelerated progression of coronary artery disease [22,23]. 

The use of a low-protein diet (LPD) in CKD was first suggested by Beale in 1869 [24], but only in 1926 did Smith try to experimentally evaluate LPD in humans, using a case study approach [25]. Later, Giordano and Giovannetti showed that an LPD, supplemented with essential amino acids, was effective in reducing uremic symptoms [26].

Although much research has been published since 1960 regarding the impact of LPD in persons with CKD [27,28,29,30], the largest study, The Modification of Diet in Renal Disease (MDRD) study, was published in 1994 by Klahr et al. [9]. This study evaluated the impact of blood pressure control and protein restriction upon CKD progression and included two randomized controlled trials (RCTs) with a mean follow-up of 2.2 y (average age of 52 y; 60% men). In RCT 1, 585 patients (moderate kidney disease, GFR 25–55 mL/min/1.73 m^2^ of body-surface area) were randomly allocated to an usual protein diet or an LPD group (1.3 or 0.58 g of protein/kg of standard body weight/day and 16–20 or 5–10 mg phosphorus/kg of standard body weight/day, respectively) and to an usual or a low-blood-pressure group (mean arterial pressure of 107 or 92 mm Hg, respectively). In RCT 2, 255 patients (advanced kidney disease, GFR of 13–24 mL/min/1.73 m^2^ of body-surface area) were randomly enrolled to an LPD or a very low-protein diet (VLPD) group (0.58 or 0.28 g of protein/kg of standard body weight/day and 5–10 or 4–9 mg phosphorus/kg of standard body weight/day, respectively) with a keto acid-amino acid supplement and an usual or a low-blood-pressure group (same values as those in RCT 1). The authors concluded that an LPD offers a small benefit only in patients with moderate renal insufficiency and that a VLPD, *versus* LPD, did not slow the progression of CKD among patients with more severe renal insufficiency. Importantly, with the exception of the group with an usual protein intake, which achieved a lower value than prescribed (1.11 g of protein/kg of standard body weight/day *versus* 1.3 g of protein/kg of standard body weight/day), all the other groups achieved a higher protein intake. In RCT 1, the LPD group had a mean protein intake of 0.77 g of protein/kg of standard body weight/day, and in RCT 2, the LPD group achieved a mean protein intake of 0.73 g of protein/kg of standard body weight/day, while the VLPD group, supplemented with ketoacids and amino acids, achieved a mean protein intake from food of 0.48 g of protein/kg of standard body weight/day. No differences were observed regarding adverse effects [9]. Secondary analysis of MDRD results and additional research concluded that, taken together, the evidence seems to support the efficacy and safety (with good planning) of an LPD (0.58 g of protein/kg of body weight/day and 5–10 mg phosphorus/kg of standard body weight/day) in CKD, particularly under strict blood pressure control (at least 80% of patients in RCT 1 and 85% of patients in RCT 2 used antihypertensive drugs during more than half of the follow-up period to achieve mean arterial pressure goals) [31].

The rationale for using an LPD is the avoidance of glomerular hypertension, which leads to glomerular hyper-filtration with an increased nephron workload. A high-protein diet contributes to hyper-filtration, with increased hemodynamic stress and production of growth factors and cytokines, lower tone of afferent arteriole, and glomerular membrane perm-selectivity. In addition, single-nephron hyper-filtration increases protein leakage and intra-glomerular pressure, leading to tubulointerstitial fibrosis and glomerulosclerosis, which are crucial contributors to CKD progression that may be worsened by metabolic acidosis [32,33].

The National Kidney Foundation’s Kidney Disease Outcomes Quality Initiative (KDOQI) states that under sufficient energy intake (>30 Kcal/kg of body weigh/day), a protein intake of 0.55–0.60 g/kg of body weight/day can be safe and that a further restriction, plus keto-acid analogue supplementation, may guarantee a sufficient balance of essential amino acids. In this regard, for adults with CKD stages 3–5 not on dialysis and without diabetes who are metabolically stable and under close clinical supervision, KDOQI guidelines recommend a protein restriction of (a) 0.55–0.60 g of dietary protein/kg of body weight/day with or without keto-acid analogues or (b) 0.28–0.43 g of dietary protein/kg of body weight/day with additional keto-acid analogues to achieve 0.55–0.60 g of protein/kg of body weight/day. In addition, in adults with stages 3 to 5 CKD not on dialysis and with diabetes, the KDOQI recommends a dietary protein intake of 0.60–0.80 g/kg of body weight/day to maintain a stable nutritional status and optimize glycaemic control [10]. An important commentary by the International Society of Renal Nutrition and Metabolism (ISRNM) on the KDOQI guidelines highlights that clinicians may consider a target of 0.60–0.80 g of protein/kg of body weight/day, independently of CKD aetiology, while striving to achieve protein ingestion values closer to 0.60 g/kg of body weight/day. A VLPD may be challenging to achieve because keto-acid analogues and/or dietitians may not be available, particularly in middle- and low-income countries where CKD is increasing [34].

Since the publication of the 2020 KDOQI guidelines, two systematic reviews have been published regarding the use of LPDs for non-diabetic [11] and diabetic kidney disease [12]. In the first systematic review, Hahn, Hodson, and Fouque [11] included RCTs and quasi-RCTs (total of 17 studies, some with vegetable protein) in which 2796 adults with non-diabetic CKD (stages 3–5) not on dialysis were randomized to receive a VLPD (defined as 0.30 to 0.40 g of protein/kg of body weight/day) compared with an LPD (defined as 0.50 to 0.60 g of protein/kg of body weight/day) or an LPD compared with a normal protein intake (≥0.80 g/kg of body weight/day) for at least 12 months and up to 50 months (25–40 Kcal/kg of body weight/day). Among 10 studies comparing an LPD with a normal protein diet, mainly in CKD categories 3a and 3b, they observed a small or no difference in the numbers of participants who died (moderate-certainty evidence) or who reached ESKD (low-certainty evidence) compared with a normal protein diet. It is not determined yet whether an LPD *versus* a normal protein intake modulates the final GFR or changes in GFR (very low-certainty evidence). Eight studies compared a VLPD with an LPD, and two studies compared a VLPD with a normal protein diet in CKD (stages 4–5). A VLPD *versus* an LPD made little or no difference in terms of death but reduced the number of participants who reached ESKD (moderate-certainty evidence). Thus, it is still undefined whether a VLPD *versus* an LPD or a normal protein intake modulates the final GFR or changes in GFR (very low-certainty evidence). In addition, regarding the impact of protein restriction on body weight and nutritional status, only three studies reported participants’ final body weights, so it is undetermined whether the intervention modified the final body weight. Twelve studies disclosed no sign of PEW, while three studies described small numbers of participants in each group with PEW. Importantly, in most studies, satisfactory diet compliance was observed. In the second systematic review, Jiang, Fang, and Li [12] included RCTs and quasi-RCTs (total of eight studies) in which 486 adults (49–90% men; mean age of 33–58 y) with diabetic CKD not on dialysis were randomized to receive either an LPD (0.60 to 0.80 g/kg of body weight/day) or an usual or unrestricted protein diet (UPD; ≥1.0 g/kg of body weight/day) for at least 12 months and up to 5 y (mean duration of 2 y). Considering the high risk of bias reported in most of the included studies, the grade of evidence was low or very low. A small or no impact upon death (358 participants from five studies) and upon the number of participants who reached kidney failure (287 participants from four studies) was described for an LPD (low-certainty evidence). Compared to an usual or UPD, it is still not clear whether an LPD reduces the decline in GFR over time (367 participants from seven studies; very low-certainty evidence) or modulates the per-year deterioration of creatinine clearance (203 participants from three studies). Regarding nutritional status, only one study (out of eight) reported malnutrition in persons following an LPD. Importantly, LPD adherence was unsatisfactory in approximately half of the studies.

Results from LPDs/VLPDs, supplemented with keto-analogues, appear to be more consistent, not only delaying the progression of CKD but also contributing to the maintenance of nutritional status [6,35,36].

In a systematic review that included 1459 participants from 16 RCTs and 1 crossover study (8 studies included a small number of diabetic patients) with a large range of CKD stages, a mean age of 42–79 y, and a follow-up duration ranging from 1 week up to 3.2 y but without data regarding the percentage of men included in the studies and diet compliance, Chewcharat et al. [35] reported that a restricted protein diet (including both an LPD and a VLPD with vegetarian and mixed types of protein), supplemented with keto-analogues, preserved the estimated GFR (eGFR) and reduced proteinuria, circulating cholesterol, phosphate, and parathyroid hormone (PTH), as well as systolic and diastolic blood pressure. Furthermore, subsequent subgroup analysis showed that (a) a VLPD with keto-analogues was probably superior to an LPD with keto-analogues in decelerating the decay in eGFR and that (b) only a VLPD with keto-analogues improved circulating PTH and systolic and diastolic blood pressure. Both interventions decreased circulating phosphate. In a systematic review and meta-analysis, Bellizzi et al. [36] enrolled 16,923 individuals with diabetic kidney disease (stages 3–5) or diabetic nephropathy from 11 studies with distinct designs (pre–post interventions and prospective and retrospective cohort studies, as well as RCTs), follow-up durations (from 3 up to 108 months), and male percentages (≥60% and <60% men in 3 and 5 studies of the studies considered within the meta-analysis, respectively). In line with the aforementioned results, these researchers found favourable effects upon renal, metabolic, clinical, and nutritional outcomes of a protein-restricted diet (an LPD or a VLPD), supplemented with keto-analogues, in diabetic non-dialysis CKD patients. Furthermore, an LPD or a VLPD, supplemented with keto-analogues, improved the uremic load, weakened the progression of CKD (through the stabilization of the GFR decline), lessened urinary protein excretion, improved the homeostasis of blood glucose, and reduced blood pressure (namely, systolic blood pressure), with no adverse effects upon circulating albumin and body weight. Both supplemented diets were safe and well-tolerated. In this systematic review, dietary compliance was assessed only in two studies, using any of the following: dietary protein intake >0.60 g/kg of body weight/day, caloric intake <30 Kcal/kg of body weight/day, or keto-analogue intake less than the prescribed dose. In addition, only one study was interventional and showed good adherence to a protein-restricted diet in 45% (for a VLPD) to 69% (for an LPD) of participants. 

In a recent retrospective cohort study of 1042 pre-dialysis CKD patients (436 diabetic patients; 32.9 months of median follow-up; mean age of 72.1 y; 53.4% men), Ariyanopparut et al. [6] demonstrated that an LPD [0.60–0.80 g of protein/kg of (ideal) body weight/day and 25–35 Kcal/kg of (ideal) body weight/day], supplemented with keto-analogues, was associated with a lower rate of eGFR decline and long-term dialysis initiation *versus* an LPD alone (with a comparable protein intake between groups), even after adjustment for age, sex, diabetes status, comorbidities, and laboratory parameters (or stratification by diabetes status for eGFR), without any impairment in nutritional status as evaluated by circulating albumin levels. In fact, circulating albumin levels increased in the LPD + keto-analogues group *versus* the LPD group, even when stratified by diabetes. Although these last results should be confirmed by RCTs, it seems plausible to consider decreased protein synthesis owing to insufficient amino acid supply and/or malnutrition a concern regarding the use of long-term protein-restrictive diets in CKD patients [37,38]. Inappropriate amino acid supply may impair renal gluconeogenesis, which seems to be associated with CKD progression [39]. Thus, keto-analogues can serve as substrates for protein synthesis, avoiding the production of deleterious nitrogen-containing waste products. Keto-analogue supplementation within a protein-restricted diet allows for a reduced intake of nitrogen and may prevent the harmful outcomes of inadequate dietary protein consumption and malnutrition [40,41,42]. However, at the moment, KDOQI guidelines, as previously mentioned, recommend keto-analogue supplementation only when associated with VLPDs [10]. 

In light of the above information, the evidence coming from RCTs concerning the use of an LPD in people with CKD not on dialysis (both diabetics and non-diabetics) to slow the progression of CKD or prevent death is not robust. In addition, LPD compliance seems difficult, particularly in people with diabetes, which may explain (or contribute to) the low level of efficacy of LPDs found in clinical trials [9,11,12]. Moreover, there is limited information on adverse effects, particularly regarding nutritional status and quality of life. However, evidence from large observational studies supports the efficacy and safety of LPDs in CKD, especially with a highly personalized nutritional approach, at least in the short term [43,44,45]. Interestingly, when evaluating the effects of LPDs/VLPDs, supplemented with keto-analogues, RCTs have found more consistent results; however, malnutrition and PEW are often not fully assessed [35,36]. Considering the metabolic disturbances commonly seen in CKD that may increase the risk of malnutrition and, among other effects, bone and mineral disorders [18], the following question remains: is there any relationship between LPDs/VLPDs and malnutrition or mineral and bone disorders?

## 3. Low-Protein Diets and Malnutrition in CKD

Malnutrition (meaning undernutrition) embraces PEW, characterized by nutritional and metabolic impairments, along with the loss of body protein and energy stores (i.e., muscle and fat mass) and micronutrient deficiencies [46]. Malnutrition has been associated with increased risk of morbidity and mortality in people with CKD [47]. Malnutrition in CKD involves an interplay between several factors, including inflammation, hormonal imbalances, increased catabolism, and decreased appetite and food intake. 

Anorexia is common in CKD patients. It most probably derives from (a) alterations in the ratio of anorexigenic to orexigenic hormones; (b) build-up of metabolic waste products; and/or (c) abnormal taste, leading to decreased appetite and food intake [47]. Gastroparesis may also compromise food intake and is a common symptom in CKD patients, particularly in the more advanced stages of the disease [48,49]. Moreover, the chronic inflammatory state in CKD, which may be potentiated through the dysbiosis of the gut microbiome [50], has been associated with an increase in the resting energy expenditure, which increases protein catabolism and decreases anabolism. In addition, metabolic acidosis ensues early in CKD and has also been associated with increased protein catabolism [47]. With respect to micronutrients, dietary restrictions and anorexia may contribute to their inadequate ingestion, while drugs (like diuretics) increase their loss. Vitamin and mineral deficiencies may contribute to anaemia, CVD, and metabolic imbalances seen in CKD [51]. Taken together, the abovementioned alterations may lead to PEW and malnutrition. Thus, controlling not only nutritional intake but also the aforementioned comorbidities/symptoms (with both nutritional and/or pharmacological interventions) may prevent PEW and malnutrition [47].

Yue et al. [15] systematically reviewed the pooled effects of 29 RCTs (17 diabetic nephropathy studies and 12 CKD studies) where 1784 individuals in the LPD arm (0.28–0.80 g of protein/kg of body weight/day) and 1782 individuals in the normal protein diet arm (≈1 g of protein/kg of body weight/day) were enrolled (without data regarding the percentage of men or women within the included studies) with follow-up ranging from 2 weeks up to 60 months (mean of 16 months) and found that long-term LPD (longer than 12 months), although positively modulating CKD (reducing urinary urea, proteinuria, and blood urea nitrogen), reduced body weight and the body mass index (BMI). Dose–response analysis showed that a decrease of 0.1 g/kg of body weight/day of protein intake was associated with a reduction of 0.088 kg/m^2^ in BMI, 0.003 mmol/L in proteinuria, 0.680 kg in body weight, and 0.271 g/L in albumin. 

Other recent systematic reviews have revealed that LPDs and VLPDs appear to have no or minimal effects upon the risk of malnutrition [11,12,36]. However, when interpreting these results, the following aspects should be considered: (a) few studies focused on nutritional status as an end point of dietary interventions; (b) distinct definitions/assessments of malnutrition were used (since in some studies, malnutrition was assessed exclusively through body weight, while others evaluated malnutrition through more complete anthropometric parameters, in addition to plasma markers, such as albumin and prealbumin, and food intake); and (c) frail, elderly patients and/or patients already at increased risk of malnutrition were often excluded from the studies selected for inclusion in systematic reviews.

Addressing the incremental protein needs of elderly people with an LPD in the setting of CKD may be challenging. As CKD is, itself, associated with loss of muscle mass (which seems to be associated with low-grade inflammation, metabolic acidosis, insulin resistance, and hormonal imbalances, among other factors), the aging process, along with body composition modification and metabolic dysfunction, may increase the risk of malnutrition in this particular population. This risk may further increase in elderly CKD patients following an LPD, particularly if not well planned or monitored [16,17].

Noce et al. [52] enrolled 41 CKD patients (27% diabetic; 54% men; mean age of 73 y) to follow a controlled protein diet (protein intake of 0.70 g/kg of ideal body weight/day, 30–35 Kcal/kg of ideal body weight/day), which was associated with a low phosphorous intake (8–12 mg/g of protein) and an individualized potassium consumption (based upon blood concentration), as well as a limited sodium and calcium intakes (less than 2 g/day for both), for 6 weeks. Although the LPD had a positive impact on kidney function (decreasing azotaemia and circulating creatinine and increasing eGFR, in association with a decrease in systolic blood pressure), negative modulations of the nutritional, inflammatory, and body composition status were found, namely (a) decreases in circulating albumin and total proteins, (b) an increase in circulating C-reactive protein, (c) reductions in body weight and BMI, (d) an increase in total body water (with the consequent impairment of fat-free mass percentage), (e) a decrease in muscular mass, and (f) progressive reductions in body cell mass and body cell mass index. In addition, the phase angle decreased, which has been associated with a worse prognosis in different conditions, including CKD [53,54].

In contrast, in a recent study, Cacciapuoti et al. [55] showed that following an LPD with an adequate energy intake (LPDAE; prescription of 0.60–0.80 g of protein/kg of ideal body weight/day and 30–35 Kcal/kg of ideal body weight/day) for 6 months did not increase malnutrition risk in older people [n = 42; 83.3% men; 88.1% hypertension; mean age of 71.5 y (≥65 y)] with CKD (stages 3–5) under conservative therapy and without risk of malnutrition at baseline (Geriatric Nutritional Risk Index ≥ 98). In fact, LPDAE-compliant patients (n = 19), unlike non-compliant LPDAE patients (n = 23, with increased protein intake and decreased energy intake), exhibited improved nutritional and body composition status, namely increased free fat mass, skeletal mass, and skeletal mass index, and decreased fat mass and waist circumference. The same occurred for several circulating biochemical parameters, including glucose, HDL cholesterol, triglycerides, urea, uric acid, phosphorus, PTH, and the triglycerides/HDL cholesterol ratio. Among the non-compliant patients, increases in circulating phosphorus and PTH, as well as an increase in systolic blood pressure, were disclosed. Regarding kidney function, eGFR did not change for either the compliant or non-compliant group. Similar results were previously obtained in older patients with CKD at risk of malnutrition and following an LPD [56]. However, meeting energy requirements may be challenging in older people with CKD, even with routinely standard dietary counselling, and may be associated with a worse nutritional status [57].

Trying to solve this dilemma, the European Society for Clinical Nutrition and Metabolism (ESPEN) and the European Renal Nutrition group of the European Renal Association (ERN-ERA) wrote a conjoint critical paper [16] reviewing and summarizing the key concepts related to prevention and treatment of both CKD progression and impaired nutritional status in older people. ESPEN and ERN-ERA state that a careful assessment should be mandatory in order to identify the most urgent clinical challenge, defining the priorities for the intervention. Protein restriction should be avoided or delayed in individuals with malnutrition/PEW, particularly in those with stable kidney function. On the other hand, under CKD progression and in advanced stages, protein restriction should be prioritised in individuals with a good nutritional status. ESPEN and ERN-ERA emphasize the importance of individual risk–benefit assessment aiming to identify the most suitable intervention that should be routinely monitored in order to be adjusted as and/or when needed to improve care and outcomes. In this regard, ESPEN and ERN-ERA reinforce the role of dietitians/nutritionists in promoting diet adherence, using personalized and tailored solutions, as LPDs may be monotonous, hindering long-term use. In fact, some research suggests that personalized nutritional interventions may not only improve/maintain kidney function and nutritional status but also quality of life [43,58,59], an issue often undervalued in clinical trials focused on LPDs/VLPDs. Furthermore, Lai et al. [60] enrolled 16 adult CKD patients (mean age of 47.8 y; 37.5% men; 25% diabetic; stages 3–4) for a 16-month personalized nutritional therapy [mean 0.70 g of (fruits and vegetables) protein/kg of ideal body weight/day, mean 27.9 Kcal/kg of ideal body weight/day, ≤1000 mg of phosphorus/day]. This intervention, under the supervision of a trained renal dietitian, was characterized by a reduced protein intake and an appropriate energy intake according to KDOQI guidelines, with a controlled intake of calcium, phosphorus, sodium, and potassium. Diet compliance was verified every 3 months by the assessment of urinary nitrogen. Lai et al. described an increase in circulating bicarbonate and vitamin D, along with a reduction in circulating phosphorus. Preservation of lean mass, even in overweight subjects in whom BMI decreased, was detected. A slight improvement in muscle strength occurred, and there was no reduction in circulating albumin or total proteins. Renal function was not impacted by the intervention, as eGFR did not change over the 12-month period, and a reduction in urinary protein excretion occurred. The flow-mediated dilation of the brachial artery and the carotid intima-media thickness (early markers of endothelial dysfunction and atherosclerosis) were also not impacted by the intervention. Importantly, in this study, personalized LPD was effective in reducing markers of inflammation (i.e., C-reactive protein and erythrocyte sedimentation rate) associated with many clinical outcomes, such as PEW [60]. In addition, inflammation in CKD patients may be potentiated through gut microbiota dysbiosis [50]. Hsu et al. [61], in evaluating the effects of an LPD *versus* a normal protein diet on the gut microbiota in CKD patients (not yet on dialysis; age > 18 y; stages 2–5; 49.4–81.7% men) within the context of a systematic review and meta-analysis that included four controlled trials and one prospective observational study, detected enrichments of *Lactobacillaceae*, *Bacteroidaceae*, and *Streptococcus anginosus*, along with the depletion of *Bacteroides eggerthii* and *Roseburia faecis*. However, the changes in the gut microbiota (predominantly at the families and species levels and relatively minimal effects on microbial diversity and richness) were not associated with relevant variations in gut-derived uremic toxins, renal function, or other clinical outcomes. 

In light of the above information, LPDs/VLPDs are unlikely to cause malnutrition in the context of energy adequacy, especially when individualized and properly monitored over time. However, older individuals and/or those with more advanced stages of CKD may be at increased risk of malnutrition, so nutritional intervention should focus on identifying clinical priorities and managing them appropriately.

## 4. Low-Protein Diets and CKD Mineral and Bone Disorders

Disturbances in mineral and bone metabolism are common in CKD, mainly as the disease progress, and are a major cause of morbidity, reduced quality of life, and mortality, particularly in relation to CVD [62,63,64]. The concept of CKD mineral and bone disorders (CKD-MBD) should be used instead of renal osteodystrophy (which exclusively defines the bone pathology associated with CKD), as it describes a broader clinical syndrome that develops as a systemic disorder of mineral and bone metabolism due to CKD, characterized by abnormalities in bone and mineral metabolism and/or extra-skeletal calcification. Therefore, CKD-BMD is associated with the following parameters: (a) abnormalities of PTH, phosphorus, calcium, and/or vitamin D metabolism; (b) impairments in bone volume, linear growth, strength, mineralization, and/or turnover; and/or (c) vascular and/or other soft tissue calcification [62]. Vascular calcification is a critical component of CKD-BMD and is closely associated with CVD, emphasizing its prognostic significance [65]. 

KDIGO 2017 guidelines [66] set the lowering of high circulating phosphorus and the maintenance of circulating calcium as priorities in the treatment of CKD-MBD. Regarding diet, KDIGO proposes dietary restriction of phosphorus (with or without pharmacologic approaches), emphasizing that the phosphorus source should be considered, as its bioavailability differs greatly depending on the source (i.e., animal, vegetable, or additives). However, dietary phosphorus restriction must not compromise adequate protein intake. In fact, in the 2017 updated guidelines, there is only one reference to LPD, i.e., “restriction of dietary protein and phosphate could be achieved with maintenance of good nutrition status with intense counselling. (…) dietary protein/phosphate restriction resulted in a significant reduction in urinary phosphate excretion when compared with a control diet”. 

Regarding CKD-BMD physiopathology, phosphorous is a prime player [67,68]. Circulating phosphorus is influenced by complex interactions between the gut and bone and renal tissues. In people with normal kidney function, phosphorus homeostasis is maintained through urinary excretion in response to increased dietary and/or circulating phosphorus [69]. However, in CKD, a progressive decrease in GFR leads to phosphorus retention. Circulating phosphorus may remain normal even in advanced stages of CKD due to compensatory increases in PTH and fibroblast growth factor 23 (FGF23)/Klotho (main hormones of phosphorus metabolism) that induce phosphaturia [63,70,71]. FGF23 exerts its biological functions via receptor binding (with Klotho as cofactor), acting mainly on the kidney, by inducing phosphorus excretion and suppressing 1,25-dihydroxyvitamin D (1,25(OH)2D) synthesis. Moreover, FGF23 acts directly on the parathyroids, decreasing PTH synthesis and secretion. However, under CKD, FGF23 levels increase progressively to compensate for phosphorus retention, but the elevated FGF23 fails to suppress PTH secretion, particularly in the setting of uraemia [72]. Thus, as CKD progresses, there is a dysregulation of the FGF23–Klotho axis, meaning that increased FGF23 levels are no longer able to enhance the excretion of phosphorus, leading to hyperphosphatemia, which further stimulates FGF23 secretion from the bone [71]. In unison, increased FGF23 levels quell 1,25(OH)2D production, promoting PTH secretion and leading to a vicious cycle. Hyperphosphatemia leads to hyperparathyroidism through the induction of hypocalcaemia, decreased formation of 1,25(OH)2D, and increased PTH gene expression. As the renal function deteriorates, hyperphosphatemia and low vitamin D favour hyperparathyroidism, which appears to occur after FGF23 rises to maladaptive levels in patients with ESKD [63,70,71]. Importantly, since FGF23 increases in the initial stages of CKD, it has been considered an early marker of CKD-BMD, and its elevated levels have also been connected to an increased risk of CVD [68,71].

As CKD-BMD is associated with hyperphosphatemia and secondary hyperparathyroidism, both pharmacologic and non-pharmacologic approaches are available to adequately control those symptoms. Dietary phosphorus restriction, intestinal phosphorus binders, vitamin D supplementation, and analogues or calcimimetic agents are among the options available to manage hyperphosphatemia and/or hyperparathyroidism [63]. In the context of non-pharmacological approaches, dietary interventions for CKD-BMD have been explored—in particular, the impact of LPDs/VLPDs [18,69], as dietary phosphorus content is generally proportional to protein intake [73].

Goto et al. [74] disclosed that an LPD (0.60–0.80 g of protein/kg of body weight/day, 10–15 mg of phosphorus/kg of body weight/day, and 30–35 Kcal/kg of body weight/day) for 4 to 6 days reduced circulating FGF23 both in early (n = 15; mean age of 45 y; 13% men) and advanced (n = 20; mean age of 66 y; 65% men) CKD. Decreased circulating phosphorous and intact PTH were observed only in the latter group (which had higher baseline values for both markers), and increased circulating 1,25(OH)2D levels were found only in the early CKD group (who had higher values at baseline).

The effect of essential amino acid keto-analogue supplementation within an LPD was evaluated by Milovanova et al. in an RCT [75]. All CKD patients (79 non-diabetic; stages 3b–4; 41 males; mean age of 40.9 y) were passed through a run-in phase 1 (1 month), where they had to follow an LPD (0.60 g of protein/kg of body weight/day, containing 0.3 g of vegetable protein and 0.3 g of animal protein, with total phosphorus content ≤ 800 mg/day and an energy intake of 34–35 Kcal/kg of body weight/day), which was followed by a run-in phase 2 (1 month) with LPD plus keto-analogues to promote LPD and LPD + keto-analogue adherence, respectively. Then, participants were randomized into two groups with a 14-month follow-up, namely an LPD + keto-analogues group (n = 42) and an LPD alone group (n = 37). In the LPD + keto-analogues group, an increase in circulating calcium occurred but without exceeding the reference values. After 14 months, (a) circulating phosphorus was higher in the LPD alone group, while circulating PTH was lower in the LPD + keto-analogues group, and (b) in the LPD + keto-analogues group, circulating FGF23 was lower, while circulating Klotho was higher *versus* the LPD alone group. Importantly, in the LPD + keto-analogues group, but not LPD alone group, nutritional status was maintained and was associated with lower mean valvular calcification, which may contribute to CVD prevention. 

A VLPD, supplemented with keto-analogues, seems to have similar beneficial effects regarding CKD-BMD [76,77,78]. 

Di Iorio et al. [76] conducted a randomized, crossover controlled trial [enrolling a total of 60 moderate-stage CKD patients (46 men; mean age of 66 y; 24 diabetic) for 18 months] and reported that a VLPD [0.30–0.50 g of protein/kg of body weight/day (animal proteins, 0 g/day; vegetal proteins, 30–40 g/day)], plus keto-analogues (mixture of essential amino acids and keto-analogues of non-essential amino acids), was more effective than a Mediterranean-like diet [0.70–0.80 g of protein/kg of body weight/day (animal proteins, 30–40 g/day; vegetal proteins, 40–50 g/day)] and a free diet [1 g of protein/kg of body weight/day (animal proteins, 50–70 g/day; vegetal proteins, 15–20 g/day)] in reducing circulating phosphorus and PTH, with a similar energy intake for all three diets (30–35 Kcal/kg of body weight/day). The same research group, also after a randomized, crossover controlled trial [including 32 CKD patients not yet on dialysis (21 men; mean age of 66 y)], described that one week of a VLPD (0.30 g of protein/kg of body weight/day, 66% vegetable protein), supplemented with a mixture of essential amino acids and keto-analogues of non-essential amino acids (*versus* an LPD with 0.60 g of protein/kg of body weight/day, 48% vegetable protein), reduced circulating FGF23 and both circulating and urinary phosphate [77]. 

Evidence for the effect of LPDs or VLPDs, with or without keto-analogues, on bone turnover markers or mineral density in CKD is scarce. Chauveau et al. [79] found that a VLPD (0.30 of vegetal protein/kg of body weight/day and 5–7 mg of phosphorus/kg of body weight/day), supplemented with essential amino acids and keto-analogues (with an energy intake close to 30 Kcal/kg of body weight/day), for 2 y was safe for 13 stable advanced chronic renal failure patients (8 men; average age of 55 y; mean GFR of 15 mL/min) regarding nutritional status and circulating PTH reduction. Although lumbar or hip site bone mass, total bone mass, and Z score decreased during the entire intervention, lean body mass increased from 6 to 24 months. 

Research on LPDs/VLPDs has also explored the potential benefits of plant-based diets as a way to reduce (animal) proteins that provide titratable acids, such as phosphorus and sulphur-containing amino acids, thereby reducing the dietary acid load [80,81]. In fact, high dietary acid load is associated with a higher risk of CKD development [82] and, in persons with CKD, is independently associated with increased risk of ESKD [83] and kidney replacement therapy, particularly in people with diabetes [84]. Although the impact of dietary acid load on bone metabolism in CKD patients is yet to be determined, a recent systematic review by Gholami et al. [85] [14 studies (11 cross-sectional) with 80,545 adult individuals without CKD, half of which enrolled only women; overall age range of 29–92 y] found no relationship between the dietary acid load [measured by both potential renal acid load (PRAL) and net endogenous acid production (NEAP)] and fracture risk. Regarding femoral and spinal bone mineral density, no association was found using PRAL, but the highest NEAP category (meaning a higher dietary acid load) was associated with lower femoral and spinal bone mineral density. 

Adherence to plant-based diets in CKD patients seems not only to benefit kidney function but also to improve some markers of CKD-BMD [86,87,88]. Moorthi et al. [89] showed the efficacy (and safety) of changing (with high adherence) from a 65% animal protein diet to a 70% plant protein diet (0.80–0.91 g of protein/kg of body weight/day and 0.80–1.3 g of phosphorus/day) in lowering urine phosphorus in 13 subjects with CKD stages 3–4 (6 men; mean age of 54.8 y; 4 diabetic) over a 4-week period, demonstrating a reduction in intestinal phosphorus absorption. In addition, this dietary intervention did not induce unfavourable changes in markers of the nutritional status of participants, namely free fat mass percentage and hand grip strength.

Results from a 15-month RCT by Garneata et al. [78] support the use and superiority of a vegetarian VLPD, supplemented with keto-analogues of essential amino acids + amino acids (0.30 g of protein/kg of dry ideal body weight/day and 30 Kcal/kg of dry ideal body weight/day), as a therapeutic intervention in CKD mineral metabolism disorders, as compared to a conventional mixed LPD (0.60 g of protein/kg of dry ideal body weight/day and 30 Kcal/kg of dry ideal body weight/day). A VLPD + keto-analogues was superior in correcting acidosis and reducing the need for bicarbonate supplementation in 207 stage 4+ CKD patients (median age of 55.2 *versus* 53.6 y and 63% *versus* 59% men, respectively). In addition, improvements in inflammation and mineral metabolism parameters, namely higher circulating calcium and lower circulating phosphates, and reductions in calcium supplementation and the proportion of patients treated with vitamin D at the end of the intervention were seen in participants in the VLPD + keto-analogues group, with no adverse reactions reported.

The potential benefits of plant-based diets in CKD may be explained by (a) an increased fibre intake, which might counteract gut microbiota dysbiosis and reduce uraemic toxin production [87,88]; (b) an increased intake of plant-derived fats, such as olive oil, with anti-inflammatory and anti-atherogenic effects [90,91]; (c) the reduced bioavailability of plant phosphorous (as compared to animal proteins), which may facilitate/improve hyperphosphatemia management [87,88]; and (d) an increased intake of micronutrients and bioactive compounds, which may favourably modulate bone metabolism [92,93].

However, a recent systematic review [94] found that evidence from clinical trials on plant-based protein interventions to maintain kidney function and prevent CKD-MBD is currently insufficient to inform clinical guidelines, warranting further investigation. The 32 included studies were considered to be of “suboptimal methodological quality” by the review authors. In addition, 27 of the 32 included studies focused upon the impact of VLPDs, with keto-analogue supplementation, while the protein source was only secondary, making it impossible to distinguish the effects of greater protein restriction from the origin of the protein. Thus, based on a subset of five studies (durations ranging from 1 week to 26 weeks, with 8 to 65 adults participants with CKD, stages 3–5), Burstad et al. [94], assessing the effects of changing protein source (animal *versus* plant-based), reported no change in kidney function in four studies and a decline in one study including patients following a plant-based diet. In addition, plant-based diets did not change serum phosphorus in three studies and reduced it in one study. Data on phosphaturic hormones, PTH, and FGF23 were limited or inconclusive.

In addition, hyperkalemia, which is common in CKD patients, can make the implementation of a plant-based diet difficult. In this regard, a recent study by Avesani et al. [95] demonstrated the feasibility and safety of a plant-based diet in 26 CKD patients (stages 4–5; not on dialysis; 65.4% male; mean age of 61 y; 6-week duration) with hyperkalemia treated with a potassium binder (sodium zirconium cyclosilicate), improving overall diet quality.

Overall, research seems to suggest that LPDs have beneficial effects on markers of CKD-MBD and that supplementation of keto-analogues may be even more beneficial. However, there are scarce data on the effects of these diets on bone turnover markers, bone mineral density, and fracture [96]. The use of vegetarian LPDs or VLPDs as an alternative to animal LPDs/VLPDs seems promising and deserving of further investigation. Moreover, it should be emphasized that some treatment strategies have been updated over the years, which may skew results. For example, (a) new drugs such as Finerenone or drugs combinations have been used to delay CKD progression and reduce mortality [97]; (b) strict assessment and intervention regarding vitamin D and the increased availability of phosphorous binders and calcimimetics have been extremely important for PTH control [98]; and (c) LPDs/VLPDs are often used in conjunction with restriction/control of potassium, phosphorus, and sodium intake (as previously mentioned). Therefore, it may be difficult to disentangle the positive results of protein-restricted diets from other concurrent therapeutic approaches that may also improve outcomes in CKD patients, some of which are related to CKD-MBD. 

Regarding the potential benefits of keto-analogue use, it should be highlighted that keto-analogues are given as salts, often using calcium, with each tablet providing 50–67 mg of additional calcium [96]. The high calcium dose may have a phosphorus-binding effect, causing a decrease in circulating phosphorus. Although there is some concern about the potentially high calcium burden of keto-analogues administered as calcium salts, which may increase the risk of calcium retention and, therefore, the risk of vascular calcification, this hypothesis was not confirmed by Milovanova et al. [75], who reported that 14 months of an LPD, supplemented with keto-analogues (*versus* an LPD alone), was associated with smaller increases in vascular stiffness, valvular calcification, and left ventricular hypertrophy. On this subject, Jamal et al., based on 11 RCTs (among the 19 studies included in their systematic review and meta-analysis) with a report on the mortality outcome [representing 4622 patients (only two RCTs with non-dialysis patients); 48–99.3% men; mean age of 47–60 y; 14.1–60.6% diabetic; 5–36 months of follow-up], showed that CKD patients assigned to non-calcium-based phosphate binders exhibited a 22% reduction in all-cause mortality compared with those assigned to calcium-based phosphate binders [99].

Moreover, lower circulating phosphorus may also be the result of reduced intake of organic sources of phosphorus, mostly coming from animal-based foods and higher intake of phytate-bound phosphorus subsequent to VLPD prescription, particularly in plant-based diets [78,96]. 

## 5. Conclusions

Protein-restricted diets, especially when supplemented with keto-analogues, could effectively improve renal end points, including the preservation of eGFR and a reduction in proteinuria and CKD-mineral bone disorder parameters without triggering malnutrition. However, close monitoring of outcomes related to nutritional status and/or mineral bone metabolism, such as body composition assessment, hand grip strength, total protein/albumin, prealbumin, *N*-terminal procollagen peptide levels, type I collagen cross-linked *C*-telopeptide, calcium, phosphates, PTH, calcitriol, calcitonin, creatinine, GFR, proteinuria, and metabolic acidosis, among others, is crucial to improve the continuous adjustment and effectiveness of LPDs and VLPDs. In addition, therapeutic approaches for CKD patients have considerably improved over time, which can make it difficult to distinguish between the positive effects of LPDs/VLPDs on outcomes regarding nutritional status and mineral bone metabolism and those of the overall optimized intervention strategy. Importantly, adherence to LDPs/VLPDs in people with CKD can be challenging, making diet personalisation and monitoring over time essential, not only to increase adherence but also to maintain nutritional status and delay CKD progression and comorbidities. In this way, the use of plant-based LPDs/VLPDs seems an interesting approach, deserving further well-designed research. Particular attention needs to be paid to the most vulnerable people with CKD, namely the elderly, those in more advanced stages, and those in middle- and low-income countries who may not have easy access to keto-analogues and/or specialised renal nutrition advice over time. In these situations, the risk of malnutrition and CKD-related metabolic abnormalities, such as CKD-BMD, may be increased.
